# *Drosophila melanogaster* Mutated in its *GBA1b* Ortholog Recapitulates Neuronopathic Gaucher Disease

**DOI:** 10.3390/jcm8091420

**Published:** 2019-09-09

**Authors:** Or Cabasso, Sumit Paul, Orly Dorot, Gali Maor, Olga Krivoruk, Metsada Pasmanik-Chor, Mina Mirzaian, Maria Ferraz, Johannes Aerts, Mia Horowitz

**Affiliations:** 1 School of Molecular Cell Biology and Biotechnology, Faculty of Life Sciences, Tel Aviv University, Ramat Aviv 69978, Israel; 2 Blavatnik Center for Drug Discovery, Tel Aviv University, Ramat Aviv 69978, Israel; 3 Department of Pathology, Brigham and Women’s Hospital, Harvard Medical School, Boston, MA 02115, USA; 4 Bioinformatics Unit, Faculty of life Science, Tel Aviv University, Ramat Aviv 69978, Israel; 5 Leiden Institute of Chemistry, Leiden University, 9502 Leiden, The Netherlands

**Keywords:** Gaucher disease, glucocerebrosidase, GlcCer, GlcSph, inflammation, unfolded protein response

## Abstract

Gaucher disease (GD) results from mutations in the *GBA1* gene, which encodes lysosomal glucocerebrosidase (GCase). The large number of mutations known to date in the gene lead to a heterogeneous disorder, which is divided into a non-neuronopathic, type 1 GD, and two neurological, type 2 and type 3, forms. We studied the two fly *GBA1* orthologs, *GBA1a* and *GBA1b*. Each contains a Minos element insertion, which truncates its coding sequence. In the *GBA1a^m/m^* flies, which express a mutant protein, missing 33 C-terminal amino acids, there was no decrease in GCase activity or substrate accumulation. However, *GBA1b^m/m^* mutant flies presented a significant decrease in GCase activity with concomitant substrate accumulation, which included C14:1 glucosylceramide and C14:0 glucosylsphingosine. *GBA1b^m/m^* mutant flies showed activation of the Unfolded Protein Response (UPR) and presented inflammation and neuroinflammation that culminated in development of a neuronopathic disease. Treatment with ambroxol did not rescue GCase activity or reduce substrate accumulation; however, it ameliorated UPR, inflammation and neuroinflammation, and increased life span. Our results highlight the resemblance between the phenotype of the *GBA1b^m/m^* mutant fly and neuronopathic GD and underlie its relevance in further GD studies as well as a model to test possible therapeutic modalities.

## 1. Introduction

Gaucher disease (GD), a lysosomal storage disorder, results from mutations in *GBA1*, the gene encoding lysosomal acid β-glucocerebrosidase (GCase). Deficient activity of GCase leads to lysosomal accumulation of glucosylceramide (GlcCer) [1], mainly in monocyte derived cells, and glucosylsphingosine (GlcSph) secretion and its accumulation in heart, kidney, liver, spleen and brain [2,3,4,5].

More than 700 mutations are known to date in the *GBA1* gene (300 mutations are published [6] and 739 mutations appear in the gnomAD browser (https://gnomad.broadinstitute.org) resulting in diverse symptoms. Therefore, GD was divided into three different clinical types: non-neuronopathic, type 1 GD, and the neuronopathic GD (nGD) forms, known as type 2 and type 3 GD [1]. Type 1 GD is the most prevalent metabolic disease among Ashkenazi Jews [7,8]. Type 1 GD patients have significantly higher propensity to develop Parkinson’s disease (PD) in comparison to the non-GD population [9] (for a review, see [10]). Type 2 GD is a devastating neuronopathic form of the disease, which results in premature death at the first years of life, while type 3 patients develop a neurological disease at later ages, with a longer life expectancy compared to type 2 patients. It is of note that a complete ablation of *GBA1* expression in humans is not compatible with postnatal survival [11].

GCase is synthesized on endoplasmic reticulum (ER) bound polyribosomes and following proper folding in the ER it is transported to the lysosomes [12]. Differently from the normal enzyme, the mutant GCase molecules are recognized as misfolded and are ER retained for folding attempts. Failure to correct misfolding leads to their ER associated degradation (ERAD). This in turn leads to ER stress, which induces the UPR machinery [13,14].

**Figure 1:**
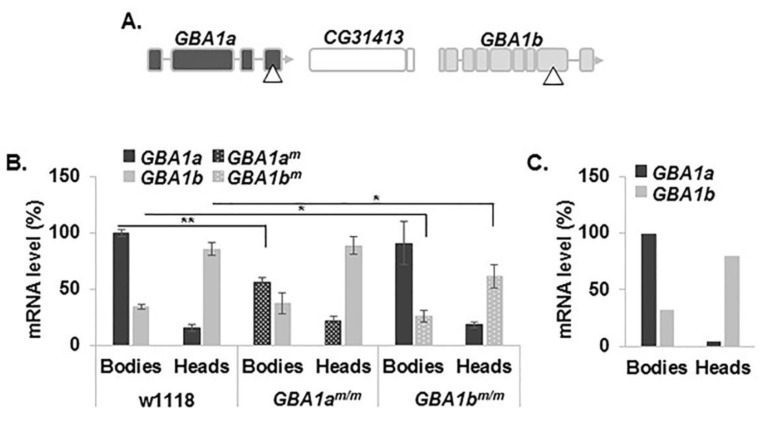
Expression of the two normal and the two mutant *Drosophila GBA1* genes. (**A**) Schematic representation of the *GBA1* genes locus. *GBA1a* is located 2 kb upstream of *GBA1b*. CG31413 is a non-relevant gene between them. Triangle represents the Minos element insertion site in each gene. Exons of *GBA1a* appear in dark grey and those of *GBA1b*-in light grey. (**B**) Expression of normal (*GBA1a* and *GBA1b*) and mutant (*GBA1a^m^* and *GBA1b^m^*) *GBA1* alleles in bodies and heads of control (w1118), *GBA1a^m/m^* and *GBA1b^m/m^* flies as analyzed by quantitative Real Time-PCR (qRT-PCR). Presented is the average ± standard error of five independent experiments. Expression of *GBA1a* in w1118 was considered 100%. * *p* < 0.05, ** *p* < 0.01. (**C**) FlyBase deep sequencing data summary (expression by tissue). Only the two highest expressed exons were counted.

To investigate the biochemical processes underling GD, a growing number of mouse models were generated over the years. Most of them are *GBA1* KO mouse (for a review, see [15]). These models contributed to the understanding of the consequences of substrate accumulation. However, the effect of ER stress due to presence of misfolded GCase cannot be studied in these models since they do not express mutant GCase. Several KI models have been developed as well; however, none of them recapitulate the human phenotypes of the parallel genotypes (for a review, see [15]). We investigated mutant flies as possible valid models for GD. There are two *GBA1* orthologs in *Drosophila* known as *GBA1a* (CG31148) and *GBA1b* (CG31414). They are ~2 and ~4 kb in size, respectively; occupy the same locus on chromosome 3 (3R: 23,700,621–23,702,605; and 3R: 23,704,804–23,708,512, respectively); and are separated by a non-relevant gene (CG31413) (Figure 1A) (FlyBase.org).

Three groups have already generated KD or KO *Drosophila* models for GD, to study the association between GD and PD [16,17,18]. Davis et al. [16] produced a fly line with endogenous deletion in the *GBA1b* ortholog (*GBA1b* KO mutant). The mutant flies showed shortened lifespan, locomotor and memory deficits, neurodegeneration, and dramatically increased accumulation of ubiquitinated protein aggregates which indicated an autophagy disruption. Ectopic expression of human alpha-synuclein in *GBA1b* KO mutants did not substantially enhance mutant fly phenotypes, except for a mild increase of dopaminergic neuron loss. Another group created a *GBA1b* deletion and a combined *GBA1a* deletion with a nonsense mutation in the *GBA1b* initiation codon, thus preventing expression of any fly GCase [17]. These flies exhibited substrate accumulation (C16:0 GlcCer), an autophagy defect, downregulation of mTOR signaling with an upregulation of the fly ortholog of TFEB, Mitf, a master regulator of lysosomal function and biogenesis [19,20]. Another study used RNAi methodology to silence the *GBA1a* gene and documented exacerbation of locomotor dysfunction, loss of dopaminergic neurons and retinal degeneration of α synuclein-expressing flies, which was associated with accumulation of proteinase K-resistant α synuclein [18]. In all the mentioned studies, mutant *GBA1* product was not present, thus they did not fully recapitulate the complexity of GD. Kawasaki et al. [21] used two existing fly lines, each containing a Minos transposable element (MiET) insertion in one of the fly *GBA1* orthologs (referred as *GBA1a^m/m^* and *GBA1b^m/m^* in the present study). Insertion of Minos in the *GBA1a* gene truncated the 561 aa coding sequence 33 amino acids upstream of its C-terminus (Figure 1A). The Minos element insertion in the 566 aa, *GBA1b*-encoded GCase, led to a C-terminal 133 aa deletion. This group showed that *GBA1b^m/m^* flies accumulated hydroxy-GlcCer and presented abnormal climbing ability, disrupted sleep and shortened lifespan.

Aiming at testing the validity of the fly as a model for GD, we followed known GD hallmarks in the two existing *GBA1* mutant lines (*GBA1a^m/m^* and *GBA1b^m/m^)*. While *GBA1a^m/m^* flies did not present any known biochemical or pathological signs of GD, *GBA1b^m/m^* flies imitated neuronopathic GD, which included massive substrate accumulation, changes in lysosomal morphology and UPR activation, inflammation, neuroinflammation, locomotion dysfunction and a significantly shortened life span. UPR activation and inflammation could be ameliorated by treatment with the pharmacological chaperone ambroxol.

## 2. Materials and Methods

### 2.1. Antibodies

The following primary antibodies were used in this study: mouse monoclonal anti-myc antibody (1:1000 for Western blotting (WB); Cell Signaling Technology, Inc., Denver, MA, USA); mouse monoclonal anti-actin antibody (1:1000 for WB, Sigma-Aldrich, Rehovot, Israel); rabbit polyclonal anti-Erk antibodies (Santa Cruz Biotechnology, Santa Cruz, CA, USA); and rabbit polyclonal anti-GlcCer antibodies (1:50 for confocal imaging; Glycobiotech, Kukels, Germany). Secondary antibodies used were: Alexa fluor 633 conjugated goat anti-rabbit antibodies (1:250 for confocal imaging; Invitrogen, Eugene, OR, USA); horseradish peroxidase-conjugated goat anti-mouse antibodies (1:5000 for WB, Jackson ImmunoResearch Laboratories, West Grove, PA, USA); and horseradish peroxidase-conjugated goat anti-rabbit antibodies (1:10,000 for WB, Jackson ImmunoResearch Laboratories, West Grove, PA, USA).

### 2.2. Construction of Plasmids

The pUASTmycHis-GBA1b^m^ and pUASTmycHis-GBA1a^m^ plasmids were constructed via Gibson assembly (New England Biolabs, Beverly, MA, USA). The truncated fragments to be cloned were obtained by High Fidelity PCR from the template pUASTmycHis-GBA1b and pUASTmycHis-GBA1a plasmids, respectively, that existed in the lab (Olga Krivoruk and Mia Horowitz, unpublished). The truncated fragments encoding the 561 aa GBA1a-encoded GCase and 566 aa GBA1b encoded GCase. The amplified fragments were inserted within pUASTmycHis [13].

To create plasmids expressing myc-tagged, normal and mutant *Drosophila GBA1b* variants in mycHispcDNA4 plasmid (Invitrogen Life-Technologies, Carlsbad, CA, USA), pUASTmycHis-GBA1b and pUASTmycHis-GBA1b^m^ plasmids were digested with EcoRI and XhoI and the myc-His containing inserts were cloned between the EcoRI and the XhoI sites of pcDNA4.

All primers used for the PCR reactions are detailed in Table 1.

### 2.3. Cells and Transfection

HEK293T cells (ATCC^®^ CRL-11268™) were grown in Dulbecco’s Modified Eagle’s Medium (DMEM; Gibco BRL, Waltham, CA, USA), supplemented with 10% FCS (Beit-Haemek, Israel) at 37 °C in the presence of 5% CO_2_. Cells were transfected using calcium phosphate solutions, as described elsewhere [22].

### 2.4. Fly Strains

All experiments were performed in isogenized w1118 background (which was also used as a control) (Bloomington *Drosophila* Stock Center, Indiana University, Bloomington, Indiana, USA). Strains harboring a Minos transposable element in *GBA1a* (Mi{ET1}CG31148) or *GBA1b* (Mi{ET1}CG31414) were obtained from Bloomington Stock Center (Nos. 23602 and 23435, respectively). The balanced lines used in this study were: w1118;Sco/Cyo;*GBA1a^m^*/TM6b,Sb **(***GBA1a^m/+^)* or w1118;Sco/Cyo;*GBA1a^m^*/*GBA1a^m^* (*GBA1a^m/m^)*; and w1118; Sco/Cyo; *GBA1b^m^*/TM6b;Sb (*GBA1b^m/+^)* or w1118;Sco/Cyo; *GBA1b^m^*/*GBA1b^m^* (*GBA1b^m/m^)*. Transgenic flies, expressing the following myc-His-tagged GCase variants were established by BestGene (Chino Hills, CA, USA) using the pUAST plasmids with the corresponding cDNAs (described under: construction of plasmids): normal human GCase, and fly *GBA1a*, *GBA1a^m^*, *GBA1b*, and *GBA1b^m^* encoded GCases. Da-GAL4 driver line was from Bloomington Stock Center (No. 55849). Strains were maintained on standard cornmeal–molasses medium at 25 °C.

### 2.5. MG132 (Carbobenzoxy-L-leucyl-L-leucyl-L-leucinal) Treatment

HEK293T cells were treated with 15 mM of MG132 (Calbiochem, San Diego, CA, USA) for 20 h.

### 2.6. Ambroxol Treatment

Eighty microliters of 1 mM ambroxol (Sigma Aldrich, Rehovot, Israel) were poured on top of 10 mL food containing vials, which were kept at room temperature for at least 1 day.

### 2.7. RNA Preparation

For RNA extraction from flies, adult flies were frozen in liquid nitrogen and then homogenized in TRIzol^®^ Reagent (Life Technologies, Carlsbad, CA, USA), according to the manufacturer’s instructions. For RNA extraction from hemolymph, flies were anesthetized, decapitated and perforated at their thorax, using insect pins (0.15 mm, Fine sciences tools, Heidelberg, Germany). The flies were collected in 0.2 mL PCR tubes. Each PCR tube contained no more than 50 flies. A small aperture was made at the bottom of the PCR tube with a needle, after which it was placed in a 1.5 mL Eppendorf tube and centrifuged at 10,000× *g* for 10 min at 4 °C. The hemolymph was collected in the 1.5 mL Eppendorf tubes. RNA was isolated from the hemolymph by standard procedure using TRIzol^®^ reagent.

### 2.8. RT-PCR

One microgram of RNA was reverse-transcribed with MMLV reverse transcriptase (Promega Corporation, Madison, CA, USA), using oligo dT primer in a total volume of 25 µL, at 42 °C for 60 min. Reactions were stopped by incubation at 70 °C for 15 min.

### 2.9. Quantitative Real Time PCR (qRT-PCR)

Two microliters of cDNA were used for real time PCR. PCR was performed using “power SYBR green QPCR mix reagent” kit (Applied Biosystems, Foster City, CA, USA), by Rotor-Gene 6000. The reaction mixture contained 5 µL of SYBR green mix, 300 nM of forward primer and 300 nM of reverse primer, in a final volume of 10 µL. Thermal cycling conditions were: 95 °C (10 min), and 40 cycles of 95 °C (10 s), 60 °C (20 s) and 72 °C (20 s). Relative gene expression was determined by Ct value and normalized to that of RP-49 gene. All primers used for the analyses are detailed in Table 1.

### 2.10. Whole Transcriptome Sequencing and Analysis

The Illumina NGS sequencing was performed at the Weizmann Crown institute for Genomics, Rehovot, Israel. Briefly, libraries were prepared from RNA samples, extracted from bodies and heads of 12-day-old flies using in house protocol. For each line (w1118, *GBA1a^m/+^*, *GBA1a^m/m^*, *GBA1b^m/+^* and *GBA1b^m/m^*), triplicates of fifty flies were used. Samples were sequenced on two lanes of Illumina HiSeq 2500 machine, using the Single-Read 60 protocol. The output was ~15 million reads per sample. Reads were trimmed using cutadapt ((https://cutadapt.readthedocs.io/en/stable/) and mapped to *Drosophila melanogaster* BDGP6 genome (downloaded from Ensembl genomes) using STAR v2.4.2a [23] [(https://code.google.com/archive/p/rna-star/;) default parameters]. Counting proceeded over genes annotated in Ensembl release 31 (http://metazoa.ensembl.org/Drosophila_melanogaster/Info/Index) using htseq-count (https://htseq.readthedocs.io/en/release_0.11.1/) (intersection-strict mode). Differential expression analysis was performed using DESeq2 [24] (doi:10.1186/s13059-014-0550-8) with the betaPrior, cooksCutoff and independentFiltering parameters set to False. Raw P values were adjusted for multiple testing using the procedure of Benjamini and Hochberg [25]. Pipeline was constructed using Snakemake (https://snakemake.readthedocs.io/en/v3.9.1/). Gene lists were created by filtering the genes based on an absolute linear fold change ≥2, *p* ≤ 0.05, and reads ≥30. To view gene lists as a heat map, the Morpheos tool was used (https://software.broadinstitute.org/morpheus/). Gene lists were analyzed for enriched pathways using the Gene Ontology tool (http://geneontology.org).

### 2.11. Detection of Spliced Xbp1 mRNA Processing

One microgram of RNA was reverse transcribed with MMLV reverse transcriptase (Promega Corporation, Madison, WI, USA), and was used as a template for quantitative real time PCR. The forward primer could anneal only to the spliced form of Xbp1 mRNA (primers are detailed in Table 1).

### 2.12. SDS–PAGE and Western Blotting

For each preparation, at least 20 flies were homogenized in RIPA lysis buffer (50 mM Tris/HCL, 150 mM NaCl, 1 mM EDTA, 1% TritonX-100, 1% sodium deoxycholate, and 0.1% SDS) containing protease inhibitors (10 μg/mL Leupeptin, 10 μg/mL Aprotinin and 0.1mM PMSF (Sigma-Aldrich, St. Louis, MO, USA)). Samples containing the same amount of protein were electrophoresed through 10% SDS–PAGE and electroblotted onto a nitrocellulose membrane (Schleicher and Schuell BioScience, Keene, NH, USA), which was interacted with the appropriate antibodies. The blots were developed and analyzed by ChemiDoc™ XRS (Bio-Rad laboratories, GmbH, Munich, Germany).

### 2.13. Total Lipid Extraction and Quantification of GlcCer and Ceramide

Flies were lysed in 300 µL of distilled water. Protein amount was determined by NanoDrop^®^ ND-1000 UV-Vis (Thermo Fisher scientific, Waltham, MA, USA) and 900 µL chloroform:methanol (2:1, v/v) were added. After centrifugation, the lower phase was isolated according to the Folch protocol [26] and dried. Twenty microliters of chloroform:methanol (2:1) were added and the samples were separated by Thin Layer Chromatography (TLC; Silica gel 60A; Sigma-Aldrich, St. Louis, MO, USA) in chloroform:butanol:ethyl acetate:0.25% KCl:methanol (25:25:25:9:16, by vol.). The TLC plates were developed with primulin reagent (Sigma-Aldrich, St. Louis, MO, USA) and quantified by ChemiDoc™ XRS (Bio-Rad laboratories, GmbH, Munich).

### 2.14. GlcCer and GlcSph Determination by LC-MS/MS

GlcCer and GlcSph were measured as previously described by a modification of the Bligh and Dyer method [27,28,29,30]. Briefly, 25 µL of ^13^C_5_-GlcSph (0.1 µM) were added to 50 µL of fly homogenate and lipids were extracted with methanol, chloroform, and ammonium formate buffer (100 mM ammonium formate buffer pH 3.1) (1:1:0.9; v/v/v) resulting in 2 phases. The upper phase, containing GlcSph, was dried under N_2_ stream and further extracted with water/butanol (1:1; v/v) before being applied to the UPLC-MS. GlcCer (lower phase) was separated from neutral lipids (ceramides and phospholipids) by solid phase extraction using Bakerbond silica gel (SiOH) columns (J.T. Baker, VWR, Phillipsburg, New Jersey, USA). 10 µL of the internal standard C17-dh-Ceramide (20 µM) was added and the samples were deacylated in a microwave for 1 h with 500 µL of methanolic NaOH (0.1 M). Deacylated lipids were additionally extracted with water/butanol (1:1; v/v) before being applied to the UPLC-MS. Lipids were analyzed by reverse-phase liquid Chromatography using a Waters UPLC-Xevo-TQS micro and a BEH C18 column, 2.1 × 50 mm with 1.7 μm particle size (Waters Corps. Milford, MA USA). Data was processed with MassLynx 4.1 Software (Waters Corporation, Milford, MA, USA).

### 2.15. GCase Activity Assay

Frozen bodies or heads were lysed in McIlvaine’s buffer (0.1 M citric acid, pH 4.2, and 0.2 M Na_2_HPO_4_, 29:21, v:v) and protein concentration was determined by NanoDrop^®^ ND-1000 UV-Vis (Thermo Fisher scientific, Waltham, MA, USA). Tissue homogenates containing 100 µg of protein were incubated at 37 °C with 8 µM of N-[6-[(7-Nitro-2-1,3-benzoxadiazol-4-yl)amino]caproyl]-glucosylceramide (C6-NBD-GlcCer) (Avanti Polar Lipids, Alabaster, AL, USA) in a final volume of 50 µL McIlvaine’s buffer for 1 h. Reactions were terminated by addition of three volumes of chloroform:methanol (2:1). Lipids were extracted and the lower phase was separated by TLC, as described under 2.13. N-[6-[(7-Nitro-2-1,3-benzoxadiazol-4-yl)amino]caproyl]-Ceramide (C6-NBD-Cer) was identified with an authentic standard (Matreya LLC, State college, PA, USA), using Amersham imager 600 (Amersham, Buckinghamshire, United Kingdom).

### 2.16. GCase Labeling with Activity-Based Probes

Fifty micrograms of protein, extracted from bodies or heads of flies, were incubated with 500 nM of Epoxide ME569, synthesized at the Department of Bio-Organic Synthesis at Leiden University, as described elsewhere [31], in McIlvaine’s buffer at pH 5.0 (150 mM citric acid−Na_2_HPO_4_, pH 5.0) for 30 min at 37 °C. Samples were electrophoresed through 10% SDS–PAGE. Gels were developed by Typhoon FLA 9500 scanner (GE health care, Little Chalfont, UK), capturing Alexa647/Cy5, using PMT 750 V and 100 μm pixel size or by Amersham imager 600 (Amersham, Buckinghamshire, UK).

### 2.17. Endoglycosidase H (Endo-H) Sensitivity

Endo-H sensitivity was tested essentially as described elsewhere [14]. Briefly, cell lysates, containing 70 µg of total protein, were subjected to an overnight incubation with endo-H (New England Biolabs, Beverly, MA, USA), according to the manufacturer’s instructions.

### 2.18. Lysotracker and Confocal Imaging

For LysoTracker staining, adult brains were dissected in phosphate buffered saline (PBS) and immediately transferred into PBS containing 1 µM of LysoTracker Red DND-99 (Invitrogen, Eugene, OR, USA) and 1 µM of DAPI (GBI labs, Bothel, WA, USA). The brains were mounted on slides and imaged within 15 min of dissection with a Zeiss LSM510 Meta (ZEISS, Oberkochen, Germany) confocal microscope. For each sample, one control and one mutant brain were imaged side by side, with identical microscope settings and images were taken from the same brain region. Images were quantified using ImageJ software, by setting a threshold for LysoTracker and measuring the pixel intensity. For GlcCer staining, fat body from third instar larvae was dissected in cold PBS and fixed with 4% paraformaldehyde (EMS, Hatfield, PA, USA) in PBS for 15 min. Following rinsing with PBT (1× PBS supplemented with 0.3% Triton X-100), samples were reacted with rabbit anti-GlcCer antibodies for 2 h at room temperature after which secondary antibodies were added and incubated with shaking for overnight at 4 °C. After several washes with PBT, the samples were mounted on slides with a DAPI containing medium (GBI labs, Bothel, WA, USA). Slides were visualized using LSM510 Meta confocal microscope (ZEISS, Oberkochen, Germany).

### 2.19. Isolation of Hemolymph and FACS Analysis

Hemolymph was prepared from 30 flies at 12 days of age, as detailed under: “mRNA extraction” and was collected in the 1.5 mL Eppendorf tubes containing 50 µL of Schneider’s *Drosophila* Medium (Biological Industries, Beit-Haemek, Israel). Samples were centrifuged at 1100× *g* at 4 °C for 10 min, after which medium was discarded and the pellets were washed three times with cold PBS. Fifty microliters of Lysotracker^®^ Red DND-99 (Invitrogen, Eugene, OR, USA), diluted with Schneider’s *Drosophila* Medium to a final concentration of 1 µM, were added, followed by 15 min incubation at 25 °C, in the dark. Samples were washed three times with cold PBS and fixed with 4% paraformaldehyde/PBS (EMS, Hatfield, PA, USA) for 15 min at room temperature. Gallios flow cytometry (Beckman Coulter, CA, USA) was used to sort the hemocytes. LysoTracker signal was measured by side scatter using 530 nm excitation (SSC:FITC_530) and cell size was measured by forward light scatter (FSC:LinH). FlowJo software was applied for the analysis.

### 2.20. Climbing Assay

Climbing behavior of adult flies was measured using a countercurrent apparatus essentially as described elsewhere [32]. Briefly, groups of approximately 50 flies (both males and females) were given 10 min to adapt in the starting tube, which can slide along the apparatus and then 20 s to move upwards against gravity to the upper frame’s tube. The top frame of tubes was then shifted to the right so that the start tube comes into register with a second bottom tube and flies, which successfully climbed up, were tapped down again, falling into tube 2. The upper frame was then returned to the left and the flies were once again allowed to climb into the upper tube. After five runs, the number of flies in each tube was counted. For each time point, at least four cohorts from each genotype were scored. The Climbing Index (CI) was calculated using the following formula: CI (the weighted mean) =  Σ(*mn_m_*)/N. CI ranges from 1 (min) to 6 (max).

### 2.21. Survival Assay

For each fly strain, 10 vials, each containing 5 males and 5 females, were maintained on food from day one post-eclosion. Fresh food was supplied every other day and deaths were recorded. Analysis of results was performed using Excel.

### 2.22. Statistics

All results were statistically analyzed using the student *t*-test.

## 3. Results

### 3.1. Expression of the Fly Normal and Mutant GBA1 Genes

At a first stage, we tested the expression pattern of the two fly mutant *GBA1* orthologs, in flies homozygous for the Minos insertions, in comparison to expression of the normal counterparts. The Minos insertion is supposed to disrupt normal transcription, resulting in less stable mRNA. Results of qRT-PCR analysis showed that, while expression of *GBA1a* mRNA is mostly restricted to bodies, *GBA1b* is the major species expressed in heads (Figure 1B). These results are in agreement with those of the *Drosophila* database (FlyBase.org) (Figure 1C). In both mutant lines, there was ~30% decrease in the relevant mutant mRNA level. Thus, level of *GBA1a^m^* was decreased in *GBA1a^m/m^* flies while level of *GBA1b^m^* mRNA was decreased in *GBA1b^m/m^* flies pointing to reduced stability of the mutant truncated mRNAs.

**Figure 2:**
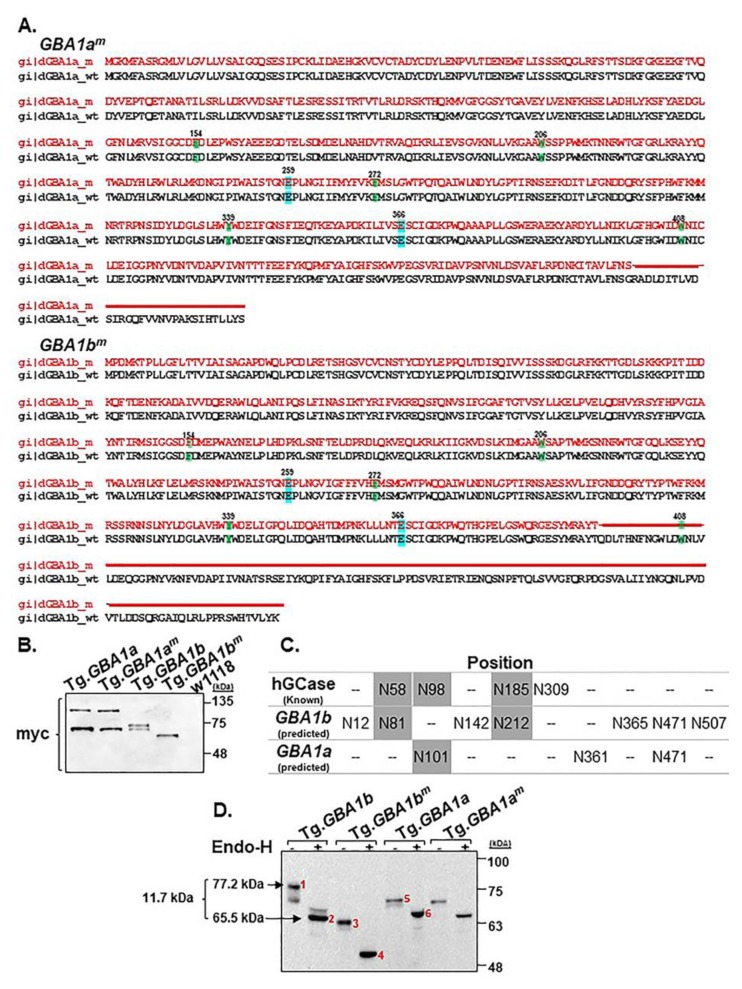
Protein products of the fly mutant alleles. (**A**) Mutant protein sequences (red) of *GBA1a* (*GBA1a^m^*) and *GBA1b* (*GBA1b^m^*) compared to the wt protein sequence (black). Red lines represent the deletion in each mutant protein (33 C-terminal amino acids in *GBA1a^m^* and 133 C-terminal amino acids in *GBA1b^m^* including W408 that stabilizes the substrate). Blue color highlights amino acids comprising the active site. Green color highlights amino acids associated with substrate recognition [33]. (**B**) Protein expression of normal and mutant *GBA1a* and *GBA1b* alleles, coupled to a myc-tag in transgenic (Tg.) flies, expressed under the daughterless (Da)-GAL4 driver. (**C**) Known glycosylation sites in human GCase and predicted ones in *Drosophila GBA1a* and *GBA1b*-encoded GCases. Common sites in fly and human GCases are highlighted with gray. (**D**) Protein lysates, prepared from transgenic (Tg.) flies expressing wt or mutant myc-tagged *GBA1a* or *GBA1b* cDNAs, under the Da-GAL4 driver, were incubated with or without endo-H. The lysates were electrophoresed through SDS–PAGE and the corresponding blot was interacted with anti-myc antibody. Peptides 1 and 2 represent *GBA1b*-encoded GCase before and after endo-H treatment, respectively. Peptides 3 and 4 represent mutant *GBA1b^m^*-encoded GCase before and after endo-H treatment, respectively. Peptides 5 (71.8 kDa) and 6 (67.4 kDa) represent *GBA1a*-encoded GCase before and after endo-H treatment, respectively.

Since *GBA1a^m^* and *GBA1b^m^* are transcribed in the mutant flies, we tested expression of the mutant proteins. It is of note that the two catalytic amino acids, which determine GCase activity, are identical between human and fly (E340 and E235 in human GCase, and E259 and E366 in fly *GBA1a* and *GBA1b* encoded GCases, respectively) (Figure 2A) [33]. The same is true for five of the six amino acids that stabilize the substrate in the active pocket of GCase (F128, W179, F246, Y313, and W381 in human GCase; and F154, W206, F272, Y339 and W408, in both fly *GBA1* encoded proteins). Amino acid W408 is missing in the mutant *GBA1b*-encoded GCase (Figure 2A). With no available antibodies, GCase expression was tested in flies expressing normal or mutant *GBA1a* and *GBA1b* transgenes. The results (Figure 2B) show the expression of the truncated proteins. *GBA1a*-encoded GCase appeared as two peptides, the upper one representing most probably a dimer that is unstable under strong reducing conditions (see Figure 2D).

The NetNGlyc 1.0 Server predicts three and seven glycosylation sites on the *GBA1a* and *GBA1b* encoded GCases, respectively (Figure 2C). Taking into account that each N-linked glycan tree, containing nine mannose residues, in the ER is ~2 kDa [34], there should be a 6 kDa and a 14 kDa increase in molecular mass of *GBA1a* and *GBA1b* encoded GCases, respectively. Glycosylation status of GCase was tested using endoglycosidase-H (endo-H) [14] (Figure 2D). The results indicate a ~11.7 kDa decrease in molecular mass of the normal and mutant *GBA1b*-encoded GCases, suggesting glycosylation of six sites (“MW analysis tool” in the Image Lab software, Bio-Rad, GmbH, Munich, Germany). There was a smaller decrease of 4.5 kDa in the molecular mass of normal and mutant *GBA1a*-encoded GCases, indicating no more than two glycosylation sites.

### 3.2. GCase Activity and Substrate Accumulation

Mutant GCase is ER-retained and undergoes ERAD. Depending on its ERAD level, only part of the mutant GCase is transported to the lysosomes. Therefore, there is decreased lysosomal GCase activity, which leads to substrate accumulation in specific cells [14].

**Figure 3:**
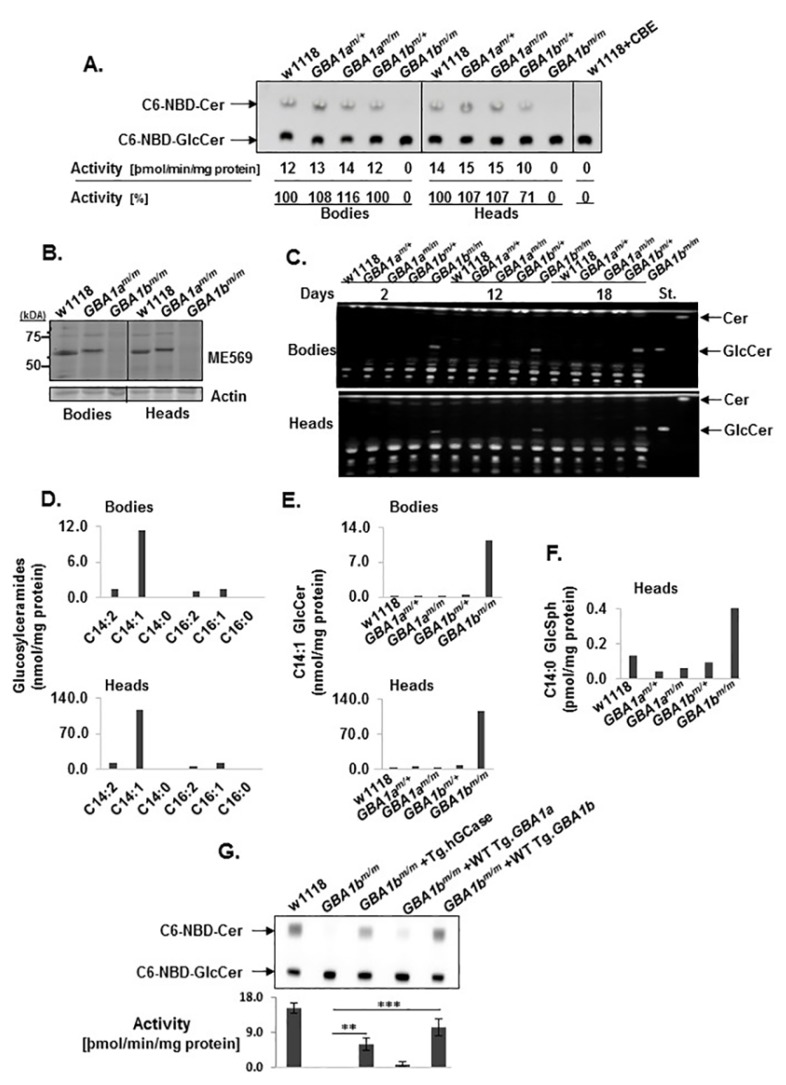
GCase activity and substrate accumulation in mutant flies. (**A**) A TLC plate presenting GCase activity as measured in lysates prepared from bodies and heads of control (w1118) and mutant flies. The value obtained for w1118+CBE (Conduritol-B-Epoxide, a GCase inhibitor) was considered zero. Shown below the plate is the average activity, obtained from three independent experiments. (**B**) ME569 epoxide ABP-labeling of active GCase in bodies and heads of control (w1118), *GBA1a^m/m^* and *GBA1b^m/m^* flies at 12 days post-eclosion. The gel was blotted with anti actin specific antibody. (**C**) TLC plates showing GlcCer accumulation in bodies and heads of 2-, 12- and 18-day-old flies. St, standards. (**D**) GlcCer species, extracted from bodies and heads of 12-day-old *GBA1b^m/m^* flies, were analyzed by LC-MS/MS. GlcCer 14:1 is the most common GlcCer species in flies. (**E**) LC-MS/MS analysis for C14:1 GlcCer extracted from bodies and heads of 12-day-old flies. (**F**) LC-MS/MS analysis for C14:0 GlcSph extracted from heads of 12-day-old flies. (**G**) Rescue of GCase activity in *GBA1b^m/m^* flies, expressing different *GBA1* orthologs as transgenes: human GCase (hGCase), *GBA1a-* or *GBA1b*-encoded fly GCases. Transgenes were expressed under the Da-GAL4 driver. Activity was tested in fly bodies as described in (**A**). Shown below the plate is the average activity, obtained from three independent experiments.

GCase activity was tested in lysates prepared from *GBA1a^m/m^* and *GBA1b^m/m^* flies, using the artificial substrate C6-NBD-GlcCer. The results (Figure 3A) indicate undetectable activity of *GBA1b^m^*-encoded GCase in heads and bodies of the *GBA1b^m/m^* flies. No reduction in GCase activity was recorded in *GBA1a^m/m^* flies. To directly test whether the *GBA1a*-encoded protein has GCase activity, we used an activity-based probe (ABP) [31]. Fluorescently labeled active-site specific probes covalently and irreversibly interact with active GCase molecules and can be followed on SDS–PAGE [31]. Since, in *GBA1b^m/m^* flies, *GBA1b*-encoded protein is inactive (Figure 3A), we tested possible *GBA1a* activity in these flies. Such analysis revealed no binding of ABP to *GBA1a*-encoded GCase (Figure 3B).

TLC analysis revealed GlcCer accumulation in *GBA1b^m/m^* flies already at Day 2 (Figure 3C). LC-MS/MS analyses indicated that C14:1 GlcCer, known as the major GlcCer species in *Drosophila* [35], accumulated in heads and bodies of *GBA1b^m/m^* flies (Figure 3D,E). There was also a notable accumulation of C14:0 GlcSph in heads of *GBA1b^m/m^* flies (Figure 3F). No substrate accumulation was noted in the *GBA1a^m/m^* flies. Since *GBA1a^m/m^* flies did not present reduced GCase activity and no substrate accumulation, and the *GBA1a^m^*-encoded GCase did not have any detectable activity toward an ABP, we abandoned further work with them. To prove that the decrease in GCase activity and substrate accumulation derive directly from the presence of mutant *GBA1b*, we tested the ability of a human or fly *GBA1* ortholog to rescue GCase activity. As shown in Figure 3G, normal human *GBA1* or fly *GBA1b* cDNAs significantly elevated GCase activity toward C6-NBD-GlcCer, while *GBA1a* encoded fly cDNA had a minor detectable activity.

### 3.3. Changes in Lysosome Morphology in GBA1b^m/m^ Mutant Flies

**Figure 4:**
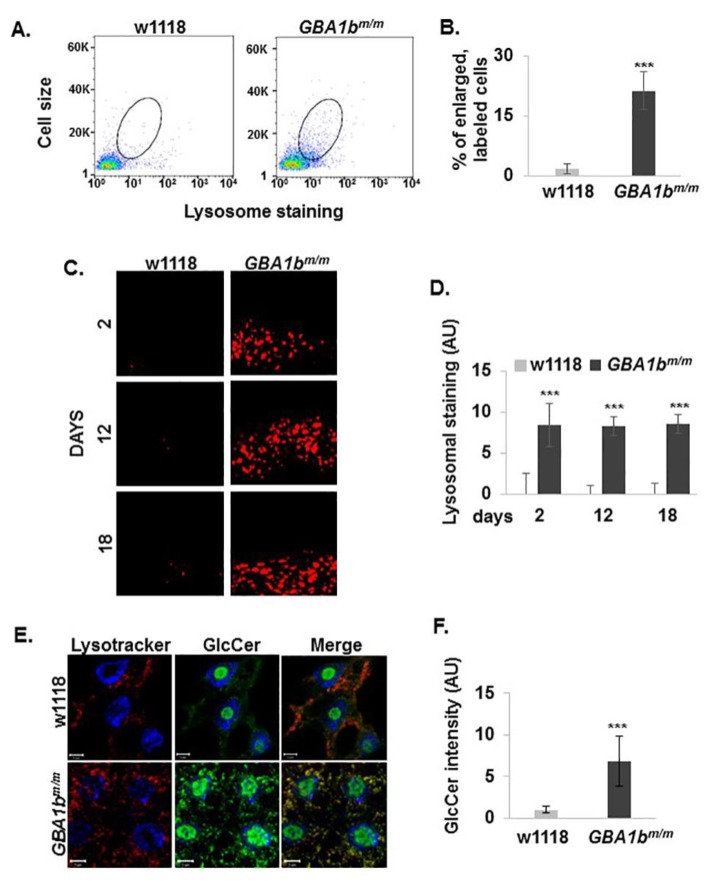
Changes in lysosomes morphology in *GBA1b^m/m^* flies. (**A**) Hemocytes were extracted from hemolymph of 12-day-old control (w1118) and *GBA1b^m/m^* flies, stained with LysoTracker-Red and analyzed by FACS. LysoTracker signal was measured by side scatter using 530 nm excitation (SSC:FITC_530) and cell size was measured by forward light scatter (FSC:LinH). LysoTracker-labeled hemocytes, larger than those extracted from w1118 flies, were encircled. (**B**). Quantification of encircled cells from (**A**). Presented is the average ± standard error of three independent assays. (**C**) Representative confocal images of the subesophageal ganglion of brains dissected from control (w1118) and *GBA1b^m/m^* flies at three different time points. (**D**) Quantification of LysoTracker in images as shown in (**C**). Presented is the average ± standard error of 30 images from each line. (**E**) Representative confocal images of Fat body dissected from third instar larvae of control (w1118) and *GBA1b^m/m^* flies, stained with Lysotracker and anti GlcCer antibodies. (**F**) Quantification of GlcCer signal as analyzed form confocal images as shown in (**E**). Presented is the average ± standard error of 30 images from each line. *** *p* < 0.0005.

Substrate accumulation in macrophage lysosomes results in their swelling [36,37]. The fly contains an open circulatory blood system, the hemolymph, which consists of three types of “blood cells”, dubbed hemocytes. Plasmatocytes represent around 90% of the hemocytes and function as macrophages to maintain cell and tissue homeostasis and to recognize pathogen entry for subsequent immune reactions. Lamellocytes are the major executioners of encapsulation during parasite infection, and crystal cells are required to stabilize wound sites and to establish a physical barrier to external microbes [38]. We tested whether the lysosomes of hemocytes in *GBA1b^m/m^* flies differ in their number and size from that of normal flies, as seen in macrophages of GD patients [39]. We did so by measuring the signal emitted from lysosomes of the fly hemocytes, using Lysotracker. The results (Figure 4A,B) strongly indicated a significant elevation both in cell size and in lysosomal signal measured in hemocytes of *GBA1b^m/m^* flies, indicating that lysosomes in hemocytes and hemocytes themselves are enlarged in comparison to those in cells isolated from w1118 control flies.

Since macrophage-like cells (plasmatocytes) comprise the major component of hemocytes [38], the documented effect represents mainly a defect in the plasmatocytes.

A previous study demonstrated a swelling of lysosomes in the subesophageal ganglion of *GBA1b* KO flies, which was not seen in age-matched control brains [17]. This phenomenon was recapitulated in the subesophageal ganglion of *GBA1b^m/m^* flies (Figure 4C,D).

To test how early substrate accumulation and lysosome swelling can be noted in the fly, fat body of third instar larva (the analog of adult human liver [40]) was stained with Lysotracker and with anti GlcCer antibodies. The results clearly show swelling of lysosomes with concomitant substrate accumulation (Figure 4E,F).

### 3.4. ERAD and UPR Activation in GBA1b^m/m^ flies

ERAD and UPR are activated as a result of expression of mutant misfolded GCase in GD [13,14]. We therefore tested whether mutant *GBA1b-*encoded GCase undergoes ERAD and elicits UPR.

**Figure 5:**
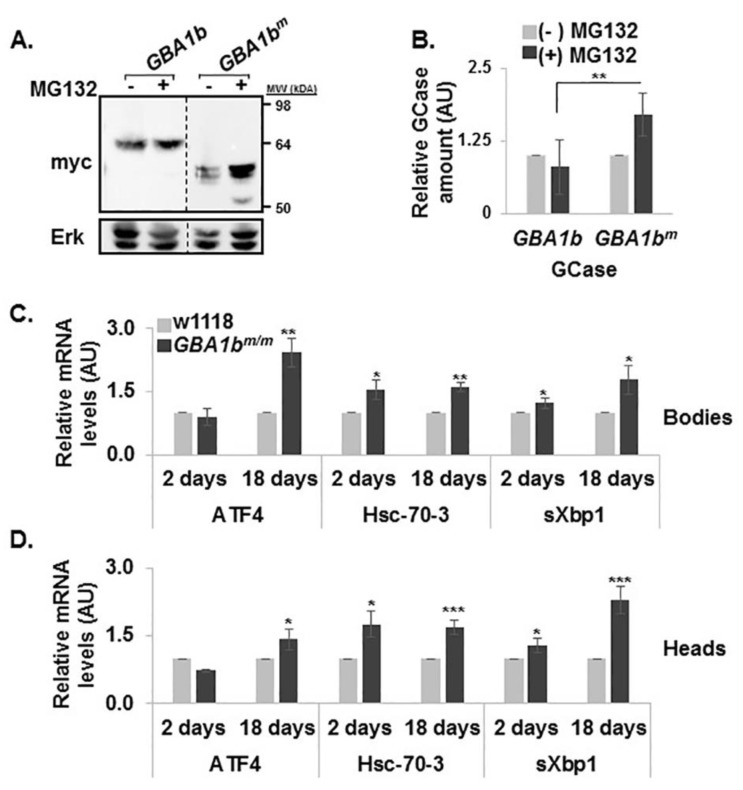
ERAD and UPR activation in *GBA1b^m/m^* flies. (**A**) Protein lysates prepared from HEK293T cells, transfected with plasmids expressing normal or mutant *GBA1b-*encoded proteins (mycHispcDNA*GBA1b*-*GBA1b*, mycHispcDNA*GBA1b^m^-GBA1b^m^*), were treated or untreated for 18 h with MG132. The corresponding blot was interacted with anti-myc and anti-Erk antibodies. (**B**) To quantify the results, WT and mutant myc-*GBA1b* intensity was divided by that of Erk in the same lane, and the number obtained for *GBA1b* without treatment was considered 1. The results represent the mean ± standard error of three independent experiments. (**C**,**D**) mRNA levels of UPR markers: activating transcription factor 4 (ATF4), Heat shock-70-3 (HSC-70-3) and spliced x-box binding protein (sXBP1), in bodies (**C**) and heads (**D**) of control (w1118), and *GBA1b^m/m^* flies at 2 and 18 days post-eclosion, as analyzed by qRT-PCR. mRNA level of each marker was normalized to that of RP49. mRNA levels of the different tested genes in control flies were considered 1. * *p* < 0.05, ** *p* < 0.01, *** *p* < 0.005.

With no available antibodies to test the different endogenous *GBA1b* encoded proteins, ERAD was tested by following the effect of a proteasome inhibitor (MG132) [41] on stability of the mutant GCase in transfected HEK293T cells. The results reveal a two-fold stabilization of the mutant *GBA1b^m^*-encoded GCase by proteasome inhibition, in comparison to the control *GBA1b*-GCase (Figure 5A,B), which imply that *GBA1b^m^* protein undergoes ERAD.

To test UPR activation, we followed the levels of the fly homolog of BiP (HSC-70-3) [42] and ATF4 [43] as well as splicing of Xbp1 mRNA [44]. The results (Figure 5C,D) indicated a significant elevation in the expression level of the three tested markers in heads and bodies of 18-day-old *GBA1b^m/m^* flies, which reflects a pathological condition triggered by expression of mutant *GBA1b^m^*-encoded GCase, as previously shown in transgenic flies expressing human mutant GCases [13].

### 3.5. Development of Inflammation and Neuroinflammation in GBA1b^m/m^ flies

**Figure 6:**
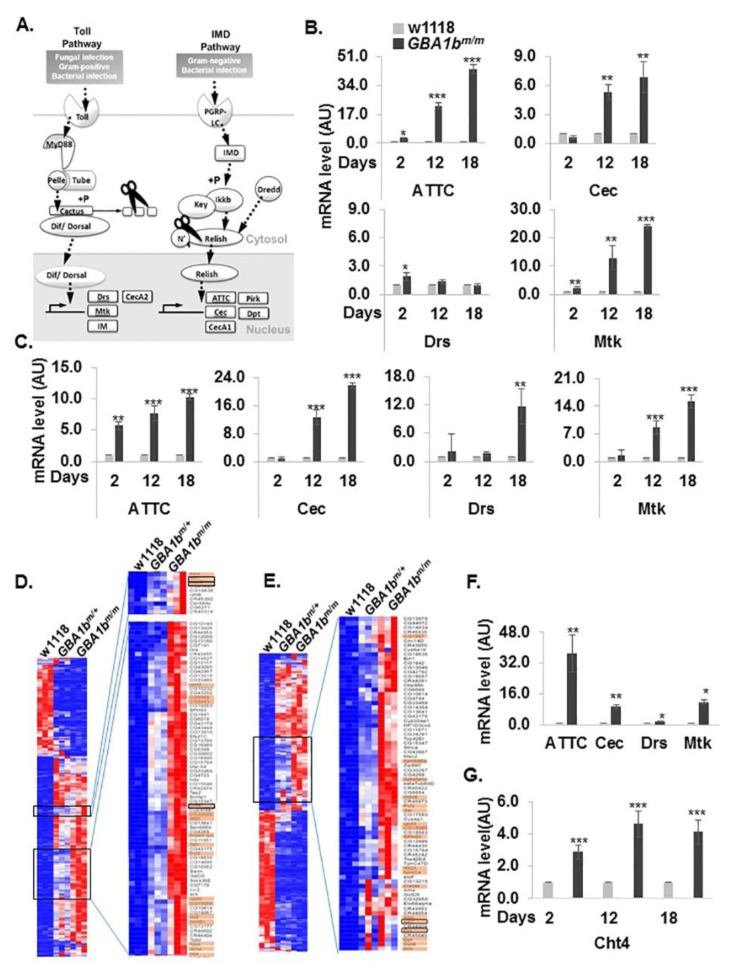
Inflammation and neuroinflammation in *GBA1b^m/m^* flies. (**A**) A schematic representation of the IMD and the Toll innate immunity pathways in *Drosophila*. (**B**,**C**) mRNA levels of Attacin-C (ATTC), Cecropin (Cec), Drosomycin (Drs) and Metchnikowin (Mtk) in bodies (**B**) and heads (**C**) of control (w1118) and *GBA1b^m/m^* flies at three different time points (2, 12 and 18 days post-eclosion), as analyzed by qRT-PCR. mRNA levels of the different tested genes in control flies were considered 1. (**D**,**E**) mRNA enrichment of gene expression in bodies (**D**) and heads (**E**) of 12-day-old *GBA1b^m/m^* flies compared to *GBA1b^m/+^* and control (w1118) flies. The right heat map is an enlargement of *GBA1b^m/m^* elevated mRNAs (boxed in the left heat map). Genes that participate in immune response are highlighted in orange. The genes analyzed by qRT-PCR in B and C are boxed. (**F**) mRNA levels of ATTC, Cec, Drs and Mtk in hemolymph of control (w1118) and *GBA1b^m/m^* flies at 12 days post-eclosion, as analyzed by qRT-PCR. (**G**) mRNA levels of chitinase 4 (Cht4) in bodies of w1118 and *GBA1b^m/m^* flies at 2, 12 and 18 days post-eclosion, as analyzed by qRT-PCR. * *p* < 0.05, ** *p* < 0.01, *** *p* < 0.005.

It is well documented that, in GD, pro-inflammatory factors, released by pathological substrate-engorged macrophages, lead to inflammation and in the brain-to neuroinflammation [45,46,47,48]. In *Drosophila*, there are two major pathways associated with immune response activation, the Toll and the Imd pathways, which are homologous to mammalian Toll-like receptor (TLR) and tumor necrosis factor receptor (TNFR) pathways, respectively (Figure 6A) [49]. Once activated, the receptors lead to signaling pathways that result in the translocation of the NF-κB homologous proteins: Dorsal in the Toll pathway and Relish in the Imd pathway, from the cytoplasm to the nucleus, where they initiate transcription of antimicrobial peptide (AMP) genes.

Each pathway is responsible for the transcription of different AMPs. To test inflammation and neuroinflammation, mRNA levels of four different AMPs, previously used to test these processes in flies [50], were measured using qRT-PCR analyses. A time dependent elevation in the mRNA levels was evident in heads and bodies of the *GBA1b^m/m^* flies, with a significant elevation of mRNA levels of ATTC, Cec and Mtk in bodies and in all four tested markers in the heads of the mutant flies (Figure 6B,C). To confirm that inflammation pathways are significantly enriched in *GBA1b^m/m^* flies, we analyzed whole exome sequencing data we have collected for 12-day-old mutant flies and their age matched controls. Bioinformatic analysis revealed that 88 inflammation-related genes in bodies and 60 inflammation-related genes in heads were significantly upregulated (Figure 6D,E), including the genes we analyzed using qRT-PCR analyses. The score values obtained for the different genes, using the Panther Gene Ontology analysis tool (http://geneontology.org/) are detailed in Table 2 and Table 3.

In *Drosophila*, the humoral responses take place mainly in the hemolymph [38]. We therefore tested whether the inflammatory markers implicated in heads and bodies are also elevated in the hemolymph of the *GBA1b^m/m^* flies. The results showed a similar pattern of elevated inflammatory markers in hemolymph of 12-day-old flies as shown in bodies (Figure 6F).

A known biomarker for macrophage activation, significantly elevated in GD patients, is chitotriosidase [51]. Bioinformatic analysis revealed that of the 10 chitinase encoding fly genes, chitinase 4 (Cht4) has 38% identity and 58% similarity to human chitotriosidase (Figure S1). Whole exome sequencing analysis demonstrated a significant elevation in the mRNA level of Cht 4 in bodies of 12-day-old flies (Figure 6D and Figure S1). This elevation was confirmed by qRT-PCR, performed with mRNA extracted from bodies of 2-, 12- and 18-day-old flies (Figure 6G).

Taken together, our results provide strong evidence for immune response activation in *GBA1b^m/m^* mutant flies.

### 3.6. Partial Rescue of the GBA1b^m/m^ Phenotype by Ambroxol

**Figure 7:**
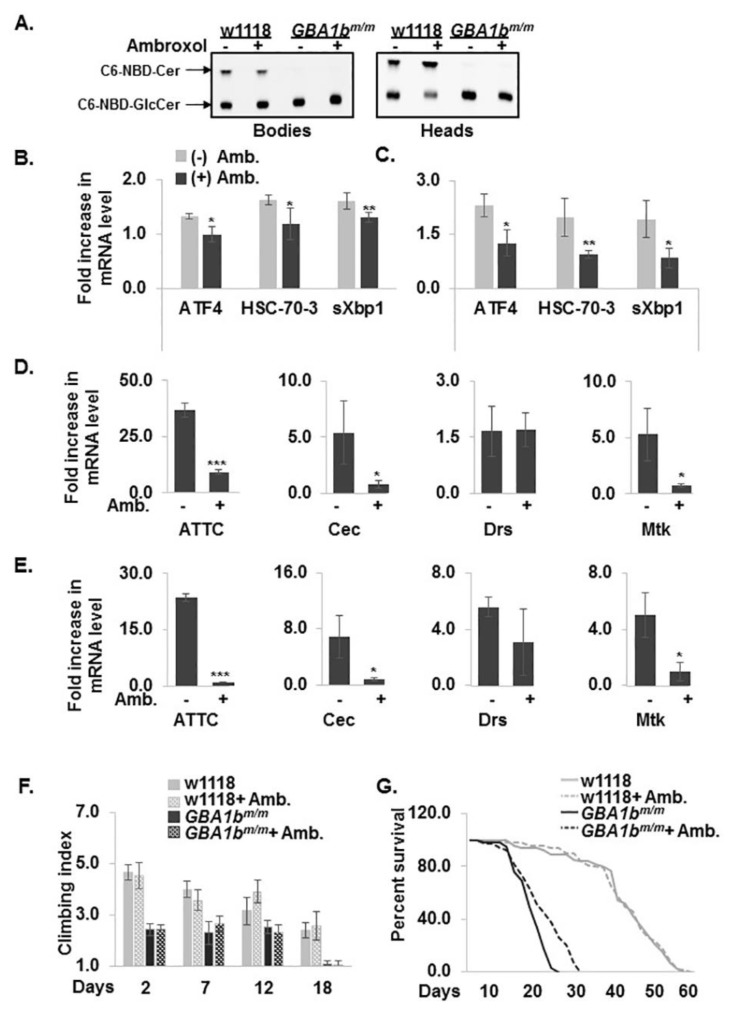
Partial rescue of *GBA1b^m/m^* pathologies by ambroxol. (**A**) A TLC plate showing GCase activity as measured in lysates prepared from bodies and heads of control and *GBA1b^m/m^* flies, treated or untreated for 12 days with ambroxol. (**B**,**C**) mRNA levels of UPR markers: ATF4, HSC-70-3 and sXBP1, in bodies (**B**) and heads (**C**) of control (w1118), and *GBA1b^m/m^* flies, untreated or treated for 18 days with ambroxol. * *p* < 0.05, ** *p* < 0.01, *** *p* < 0.005. (**D**,**E**) mRNA levels of inflammation markers: ATTC, Cec, Drs and Mtk, in bodies (**D**) and heads (**E**) of *GBA1b^m/m^* flies in comparison to age matched controls (w1118), untreated or treated for 12 days with ambroxol. mRNA levels of the tested genes in untreated control flies were considered 1. (**F**) Thirty flies from each line were untreated or treated for 12 days with ambroxol and analyzed for locomotion behavior at Days 2, 7, 12 and 18 post eclosion. (**G**) A curve showing the overall survival of w1118 and *GBA1b^m/m^* flies untreated or treated for 12 days with ambroxol. The numbers represent three independent experiments. * *p* < 0.05, ** *p* < 0.01, *** *p* < 0.005. Amb.-ambroxol.

A potential treatment that holds promise for the future of nGD patients is pharmacological chaperone therapy (PCT) [52]. One such GCase chaperone is ambroxol, initially shown to increase amount and lysosomal activity of mutant GCase in GD-derived skin fibroblasts [53,54,55] and recently shown to have an effect on neuronopathic GD patients [56]. Growth of *GBA1b^m/m^* flies on 1 mM ambroxol containing food from the day of eclosion, did not result in any change in GCase activity (Figure 7A). However, a significant reduction in UPR markers was evident in 18-day-old ambroxol-treated *GBA1b^m/m^* flies (Figure 7B,C). In addition, a significant reduction in the mRNA level of the inflammation-induced AMPs was noted in bodies (Figure 7D) and heads (Figure 7E) of ambroxol-treated 12-day-old flies, in comparison to untreated mutant flies. In parallel, we observed extension of life span of the treated mutant flies (Figure 7F). However, locomotion of the ambroxol-treated mutant flies did not change (Figure 7G), strongly indicating that the damage to fly brains due to substrate accumulation during development and adult life (as shown in Figure 4E,F) could not be reversed by ambroxol treatment post-eclosion.

Taken together, the *GBA1b^m/m^* flies, which present very low GCase activity, substrate accumulation, activation of UPR and inflammation/neuroinflammation, develop a severe neurological Gaucher-like disease. Inflammation is ameliorated and life span is increased by ambroxol. Thus, the *GBA1b^m/m^* is a relevant paradigm that imitates human GD and can serve as a model for studies of the mechanisms underlying the disease and to investigate existing and future therapeutic modalities.

## 4. Discussion

In the present study, we characterized the *GBA1b^m/m^* as a fly model for neuronopathic GD. Differently from previous studies [16,17,18], we used a fly in which its *GBA1b* ortholog was mutated. This model was also used in another study [57], which showed accumulation of hydroxy-GlcCer, defective sleep phenotype and a shortened life span. In the present work, we show that in this fly model there is expression of the mutant protein, which undergoes ERAD and elicits UPR, as we have shown in the past for GD patients [13,14]. We found that the significant reduction in GCase activity led to C14:1 GlcCer accumulation in heads and bodies of the flies and of GlcSph C14:0 in the heads of the flies. In contradiction to our results, another publication showed the accumulation of C16:0 GlcCer [17]. We noted a 30–45-fold increase in C14:1 GlcCer levels in the *GBA1b^m/m^* flies, while the previous publication showed a 7-fold increase in C16:0. In GD patients a 20–80-fold increase in the level of GlcCer was noted in comparison to control individuals [2].

GlcSph is regarded today as an important biomarker for GD [4,58]. Another marker, which is significantly elevated in GD, and decreases during treatment, is chitotriosidase [51]. Interestingly, the chitotriosidase ortholog, chitinase 4, is significantly elevated in the fly (Figure 6G). Since treatment with ambroxol does not increase activity or decrease substrate levels, this marker is not supposed to decrease in the mutant *GBA1b^m/m^* flies. It is of note that a third GD biomarker, CCL18 [45], does not exist in the fly genome.

Accumulation of excessive GlcCer in cells of the monocyte–macrophage system in GD patients results in a physical swelling of lysosomes in these cells, leading to increasing cytoplasm volume [37]. We documented a significant elevation both in cell size and in lysosomal signal in the hemolymph of *GBA1b^m/m^* flies (Figure 4). Since macrophage-like cells (plasmatocytes) comprise the majority of the hemolymph, our results strongly indicate pathology of these cells, as found in GD patients, which, most probably, results from substrate accumulation. We could recapitulate lysosomal swelling and substrate accumulation in third instar larva fat body, which is the fly homolog of human liver [40]. We also detected a massive elevation in number and size of lysosomes in the esophageal ganglion of *GBA1b^m/m^* flies, as documented by others [17]. Abnormal macrophage-like cells with large vacuoles were detected in the periventricular gray zone in brains of Medaka fish model for nGD [59].

GD is accompanied by inflammation [45,47,60] and in neuronopathic GD neuroinflammation was noted as well [46,48]. A significant elevation in IL-1β and IL-6 secretion as well as inflammasome activation were documented in macrophages derived from peripheral monocytes of type 1 GD patients [47]. Another study described splenic GD-derived cells that were positive for CD163, CD14, chitotriosidase, CD68, and HLA II [60]. Elevation in different chemokines were evident in KO [61] and KI mouse models [62]. Likewise, in a *GBA1* mutant zebrafish model for GD, microglial activation was documented in the brain [63,64]. It is plausible that inflammatory response factors, released by pathological macrophages (or glial cells), are toxic to neuronal cells and eventually lead to their death, as seen in mouse models of nGD [61]. It has already been suggested that inflammatory factors are also responsible for diverse clinical manifestations, like: hepatosplenomegaly, thrombocytopenia, destruction of bones and cell death [65]. Results of whole exome analysis and of qRT-PCR strongly indicated inflammation and neuroinflammation in the *GBA1b^m/m^* flies.

To date, available treatments for GD include enzyme replacement therapy (ERT) and substrate reduction therapy (SRT). While very efficient for type 1 GD patients, their efficacy for neurological manifestations is mostly negligible [66]. In recent years, the approach of pharmacological chaperone treatment for nGD patients is being tested [67]. The pharmacological chaperone ambroxol significantly increased mutant GCase activity in GD fibroblasts and in the cerebellum of GD mouse models [68]. Oral ambroxol administration to five type 3 Japanese GD patients (3 mg/kg/day for 6–48 months) significantly increased their lymphocyte GCase activity, decreased GlcSph levels in their cerebrospinal fluid, improved their motor function and decreased their myoclonus and seizure frequency [56]. Treatment of *GBA1b^m/m^* flies with ambroxol did not improve GCase activity and did not decrease accumulated substrate. Therefore, there was no improvement in the locomotor activity of the flies. However, a reduction in UPR markers was observed with elongation of life span. Based on hydrogen/deuterium exchange MS studies and in silico considerations [54], ambroxol binds several human GCase sequences (Figure S2) which are also present in the mutant *GBA1b*-encoded GCase. Therefore, ambroxol should bind the *GBA1b^m^*-encoded GCase. However, since *GBA1b*-encoded GCase lacks 133 C-terminal amino acids, one of which (W408) is supposed to stabilize the active site, this binding results, most probably, in shuttling of the mutant enzyme from the ER to the lysosomes, which leads to decrease in UPR markers (Figure 7B,C). However, in the lysosomes, the truncated *GBA1b*-GCase is inactive and therefore there is no increase in activity, no change in substrate accumulation and no improvement in locomotion ability of the flies. Ambroxol treatment of *GBA1b^m/m^* flies resulted in a significant reduction in inflammatory and neuroinflammatory responses. It has already been shown that ambroxol has anti-inflammatory properties [69]. Bianchi et al. demonstrated a significant reduction of IL-1 and TNF-α secretion in isolated human mononuclear cells, by the addition of 10–100 µg anbroxol/mL of medium for 24 h [70]. In another study, TNF-alpha, IL-6 and TGF-beta1 levels in the bronchoalveolar lavage were significantly reduced by ambroxol treatment of immune challenged mice [71]. In the present study, we document for the first time the effect of ambroxol on inflammation/neuroinflammation in a GD model, which is independent of its effect as a GCase chaperone. Our results indicate the additional advantage of the potential use of ambroxol for treatment of nGD patients, even without elevating GCase activity and lowering substrate levels.

To summarize, our results strongly indicate that *GBA1b-*encoded GCase is a bona fide lysosomal glucocerebrosidase. In *GBA1b^m/m^* flies, GCase activity was abolished and the flies accumulated C14:1 GlcCer in their bodies and heads as well as C14:0 GlcSph in their heads (Figure 3 and Figure 4). The enzyme itself underwent ERAD and activated the UPR machinery. Inflammation and neuroinflammation were demonstrated, all point to the development of a neuronopathic disease in the fly, imitating nGD. UPR, inflammation and neuroinflammation could be partially rescued with the pharmacological chaperone ambroxol.

Taken together, the *GBA1b^m/m^* flies seem as a bona fide model for neuronopathic GD and may be used to study the mechanisms underlying the disease and to investigate existing and future therapeutic modalities.

## Figures and Tables

**Table 1:** Primers used in the present study. The table depicts all primers used in the present study.

**Name**	**Primers for Construction of Plasmids**
***GBA1a***	F: 5′-CTGAATAGGGAATTGGGAATTCGTATGGGAAAAATGTTCGC-3′
	R: 5′-GAGGTACCCTCGAGCATTTCAAGCACTTATTGAAGAGAACGGCGG-3′
***GBA1b***	F: 5′-CTGAATAGGGAATTGGGAATTCGTATGCCAGATATGAAGACAC-3′
	R: 5′-CCCTCTAGAGGTACCCTCGAGTAGGCCCTCATATAGCTTTCACC-3′
**Name**	**Primers for qRT-PCR**
***GBA1a***	F: 5′-GAGTGGTTCCTTATCTCCAGTT-3′
	R: 5′-ACGGTAAACTTCTCCTCCTTAC-3′
***GBA1b***	F: 5′-AAGAACTTCCGGTGGAGCTA-3′
	R: 5′-CAATTCATTGTATGCCCAGGGT-3′
**sXbp1**	F: 5′-CCGAACTGAAGCAGCAACAGC-3′
	R: 5′-GTATACCCTGCGGCAGATCC-3′
**HSC-70-3**	F: 5′-GCTGGTGTTATTGCCGGTCTGC-3′
	R: 5′-GATGCCTCGGGATGGTTCCTTGC-3′
**ATF4**	F: 5′-AGACGCTGCTTCGCTTCCTTC-3′
	R: 5′-GCCCGTAAGTGCGAGTACGCT-3′
**ATTC**	F: 5′-CTGCACTGGACTACTCCCACATCA-3′
	R: 5′-CGATCCTGCGACTGCCAAAGATTG-3′
**Cec**	F: 5′-CATTGGACAATCGGAAGCTGGGTG-3′
	R: 5′-TAATCATCGTGGTCAACCTCGGGC-3′
**Drs**	F: 5′-AGTACTTGTTCGCCCTCTTCGCTG-3′
	R: 5′-CCTTGTATCTTCCGGACAGGCAGT-3′
**Mtk**	F: 5′-CATCAATCAATTCCCGCCACCGAG-3′
	R: 5′-AAATGGGTCCCTGGTGACGATGAG-3′
**RP49**	F: 5′-TAAGAAGCGCACAAAGCACT-3′
	R: 5′-GGGCATCAGATATTGTCCCT-3′

**Table 2:** Panther gene ontology analysis of fly bodies. Gene ontology enrichment analysis of genes upregulated in bodies of *GBA1b^m/m^* flies in comparison to age matched controls. Hyper genomic test was used for the calculation of fold enrichment.

Biological Process	All Known Participating Genes (*D. Mel.*)	Expected	Observed	Fold Enrichment	*p*-Value	FDR
Response to external biotic stimulus	363	1.37	18	13.13	1.03 × 10^−15^	8.04 × 10^−12^
Response to bacterium	256	0.97	15	15.57	3.88 × 10^−14^	7.56 × 10^−11^
Defense response	367	1.39	15	10.82	5.65 × 10^−12^	8.81 × 10^−9^
Defense response to Gram-positive bacterium	56	0.21	8	37.82	8.63 × 10^−11^	1.12 × 10^−7^
Response to external stimulus	912	3.44	19	5.52	4.18 × 10^−10^	4.66 × 10^−7^
Defense response to bacterium	224	0.85	11	13	8.90 × 10^−10^	8.68 × 10^−7^
Multi-organism process	1431	5.41	22	4.07	3.10 × 10^−9^	2.69 × 10^−6^
Response to stress	1122	4.24	19	4.48	1.24 × 10^−8^	8.78 × 10^−6^
Defense response to other organism	291	1.1	11	10.01	1.23 × 10^−8^	9.61 × 10^−6^
Humoral immune response	87	0.33	7	21.3	5.60 × 10^−8^	3.64 × 10^−5^
Antimicrobial humoral response	72	0.27	6	22.06	4.41 × 10^−7^	2.64 × 10^−4^
Immune response	192	0.73	8	11.03	7.30 × 10^−7^	4.06 × 10^−4^

**Table 3:** Panther gene ontology analysis of fly heads. Gene ontology enrichment analysis of genes upregulated in heads of *GBA1b^m/m^* flies in comparison to age matched controls. Hyper genomic test was used for the calculation of fold enrichment.

Biological Process	All Known Participating Genes (*D. Mel.*)	Expected	Observed	Fold Enrichment	*p*-Value	FDR
Antibacterial humoral response	28	0.09	6	68.61	7.99 × 10^−10^	6.23 × 10^−6^
Antimicrobial humoral response	72	0.22	7	31.13	4.17 × 10^−9^	1.08 × 10^−5^
Immune system process	316	0.99	11	11.14	3.24 × 10^−9^	1.26 × 10^−5^
Humoral immune response	87	0.27	7	25.76	1.42 × 10^−9^	1.59 × 10^−5^
Response to biotic stimulus	363	1.13	11	9.7	1.31 × 10^−8^	1.70 × 10^−5^
Defense response to Gram-positive bacterium	56	0.17	6	34.3	3.42 × 10^−8^	3.34 × 10^−5^
Response to bacterium	256	0.8	9	11.26	9.82 × 10^−8^	8.51 × 10^−5^
Immune response	192	0.6	8	13.34	1.58 × 10^−7^	1.23 × 10^−4^
Defense response	367	1.15	10	8.72	1.75 × 10^−7^	1.24 × 10^−4^
Defense response to other organism	291	0.91	9	9.9	2.82 × 10^−7^	1.83 × 10^−4^
Defense response to bacterium	224	0.7	8	11.43	4.91 × 10^−7^	2.95 × 10^−4^
Response to external stimulus	912	2.85	13	4.56	2.78 × 10^−6^	1.55 × 10^−3^
Response to stimulus	2640	8.25	21	2.55	1.14 × 10^−5^	5.94 × 10^−3^

## Supplementary Materials

The following are available online at https://www.mdpi.com/2077-0383/8/9/1420/s1, Figure S1: Fly chitinase 4 is the human chitotriosidase ortholog. Figure S2: Putative ambroxol binding sites in human and Drosophila GBA1-encoded GCases.
