# Supplementary material for: Drosophila melanogaster Mutated in its GBA1b Ortholog Recapitulates Neuronopathic Gaucher Disease
Source: J Clin Med. 2019 Sep 9;8(9):1420. doi: 10.3390/jcm8091420 (PMC6780790; doi:10.3390/jcm8091420)

Supplementary Materials:

The following are available online at www.mdpi.com/xxxx/s1

Figure S1: Fly chitinase 4 is the human chitotriosidase ortholog. A. BLAST analysis (https://blast.ncbi.nlm.nih.gov/Blast.cgi) of human chitotriosidase gene and the fly chitinase 4 (Cht4). **B.** Results of whole exome sequence analysis showing the fold increase in chitinase 4 level in 12 days old *GBA1b*$^{m/m}$ flies in comparison to age matched controls.

A.

```
Identities          Positives           Gaps
146/384(38%)        222/384(58%)        33/384(8%)

Human   MVLLMIPWGSAAKLV-CYFTNWAQYRQGEARFLPKDLDPSLCTHLIYAFAGMTN----HQ   65
Fly     LCLCLGQVASSEKLLNCYWGTWANYRPGDGKFTPSDIDPSLCTHISYTFFGISDAGEFKS   71

Human   LSTTEWNDETL--YQEFNGLKKMNPKLKTLLAIGGWNFGTQKFTDMVATANNRQTFVNSA   123
Fly     LDTWLDMDDGLGFISQTIALKQRNPNLKILAVVGGWNEGSTKYSAMAADPAKRATFVSTS   131

Human   IRFLRKYSFDGLDLDWEYPGSQGSPAVDKERFTTLVQDLANAFQQEAQTSGKERLLLSAA   183
Fly     LAFIQQYSFDGLDLDWEYPGQRGGSEADRENFVTLLREIKETYDQYG-------LELGIA   184

Human   VPAGQTYVDAGYEVDKIAQNLDFVNLMAYDFHGSWEKVTGHNSPLYKRQEESGAAASLNV   243
Fly     VGASEKSASISYDIPAISQHLTFINVMTYDFHMALDGYLGLNAPLPEVAE----------   234

Human   DAAVQQWLQKGTPASKLILGMPTYGRSFTLASSSDTRVGAPATGSGTPGPFTKEGGMLAY   303
Fly     --SIDYWLSHGAPAEKLILGIGFYGHSYQMSDSSQNWPGAACIGPGTAGVYTRENGFLGY   292

Human   YEVC--SWKGATKQRIQDQKVPYIFRDNQWVGFDDVESFKTKVSYLKQKGLGGAMVWALD   361
Fly     HEICLNNWQTVFD---QENGAPYAFQGDQWIGYDNPESIQLKMQLVESRNLGGAMMWSIE   349

Human   LDDFAGFSCNQGRYPLIQTLRQEL   385
Fly     TDDFRGL-CGE-SYPLLKTMNRAL   371
```

B.

	Gene	Fold-change	P-Value
Bodies	Cht4	18	6.21E-99
Heads	Cht4	2	0.799

The following are available online at www.mdpi.com/xxxx/s2

Figure S2: Putative ambroxol binding sites in human and *Drosophila GBA1*-encoded GCases. Three peptides that are stabilized through interaction of ambroxol with human GCase (according to Maegawa et. Al., 2009) are marked with ( ) on the human sequence and with ( ) on the fly encoded GCase. The amino acids AY (in purple) represent the Minos insertion site in *GBA1b*. Red labeled letters represent the 133 amino acids missing from the mutant *GBA1b*^{m} allele. W408, that stabilizes the substrate, is marked by an arrow. The indicated aa are numbered according to the mature protein (after cleavage of the signal peptide, which is marked with light blue).

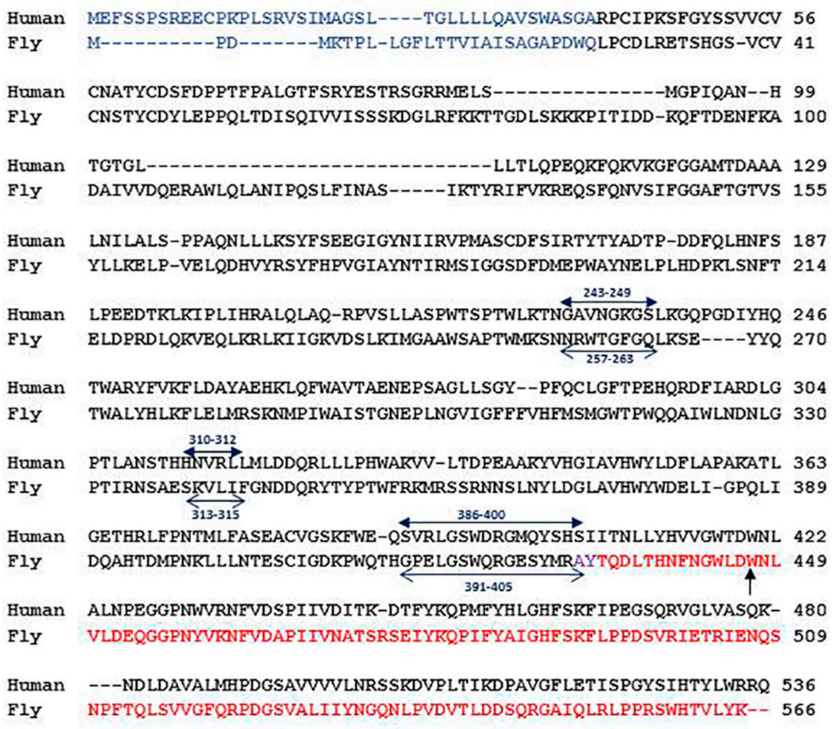

Supplement: Supplementary file 1 [file jcm-08-01420-s001.pdf]
